# Stochastic analysis of time series for the spatial positions of particles trapped in optical tweezers

**DOI:** 10.1038/s41598-017-04557-0

**Published:** 2017-07-06

**Authors:** S. M. Mousavi, S. N. Seyed Reihani, G. Anvari, M. Anvari, H. G. Alinezhad, M. Reza Rahimi Tabar

**Affiliations:** 1 Department of Physics, Sharif University of Technology, Tehran, 11155-9161 Iran; 2 Department of Bioengineering, Temple University, 1947 N 12th Street, Philadelphia, USA; 3 Institute of Physics and ForWind, Carl von Ossietzky University of Oldenburg, 26111 Oldenburg, Germany

## Abstract

We propose a nonlinear method for the analysis of the time series for the spatial position of a bead trapped in optical tweezers, which enables us to reconstruct its dynamical equation of motion. The main advantage of the method is that all the functions and parameters of the dynamics are determined directly (non-parametrically) from the measured series. It also allows us to determine, for the first time to our knowledge, the spatial-dependence of the diffusion coefficient of a bead in an optical trap, and to demonstrate that it is not in general constant. This is in contrast with the main assumption of the popularly-used power spectrum calibration method. The proposed method is validated via synthetic time series for the bead position with spatially-varying diffusion coefficients. Our detailed analysis of the measured time series reveals that the power spectrum analysis overestimates considerably the force constant.

## Introduction

Nanometer spatial resolution along with subpiconewton force resolution have turned optical tweezers (OTs) into valuable micromanipulation tools for biological and physical sciences^1–3^. The OTs are commonly used to exert or measure very accurately tiny forces^4, 5^. In a typical application, a micron-sized, mainly dielectric, sphere is used as a handle. The Hookean force experienced by the trapped bead allows for measuring unknown external forces, provided that the stiffness of the trapping force is determined prior to its use. This is typically done by a so-called calibration process. Several calibration methods have been presented^6^, among which the power spectrum (PS) method^7^ is the most utilized, due to its feasibility. The main idea behind the PS method is to analyze the time series for the spatial position of a trapped particle in the frequency domain, which endows the method with a rather unique ability. For example, mechanical or even coherent high frequency noises presented in the time series can be easily excluded by the calibration process. It is known that particles in relatively strong traps have power spectra that cannot be fitted properly with any Lorentzian function^7^.

**Figure 1:**
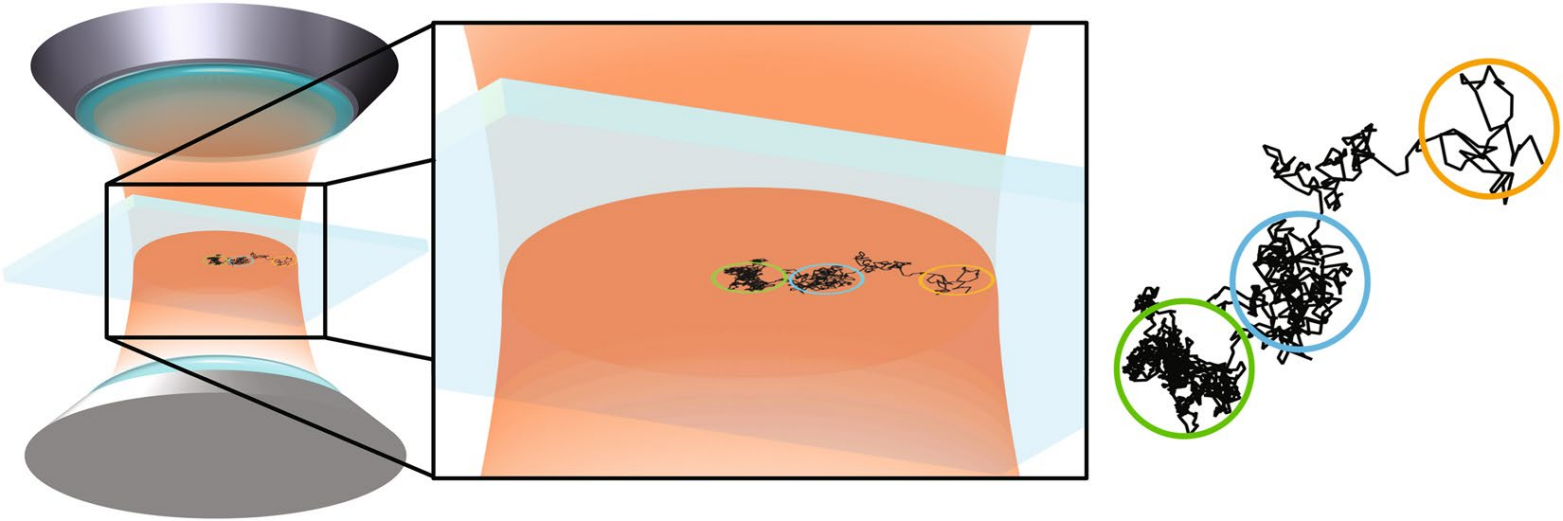
Diffusion of a particle trapped by optical tweezers. A schematic illustration of the position-dependent diffusion coefficient for a particle trapped by optical tweezers. The black kinky line is produced by connecting the positions of the bead falling into the trap. The colored circles with equal diameter are drawn at various radial distances from the center of the trap. The number of the data points inside each circle correlates with the diffusion coefficient. Thus, the larger the number of the data points, the lower is the diffusion coefficient. At the center (green circle) the particle possesses a small diffusion coefficient, whereas at larger radial distances (orange circle) it has higher diffusion coefficient.

Despite being very popular in the OT-based applications, we show in this report that SP-based calibration method is accurate only when the trapped bead is displaced by a few nanometers from the center of the trap. Considering that in the force spectroscopy applications of the OT the bead is typically displaced by hundreds of nanometers, use of the method for calibration might introduce considerable error in the measured forces. Here, we address this problem by introducing a stochastic method of analyzing the times series of the position of a trapped particle. The method is purely data driven and is based on the general properties of stochastic processes. The main advantage of the method is that, the spatial dependence of all the functions and parameters of the model, such as the stiffness and the diffusion coefficients (as shown schematically in Fig. 1) for the bead’s displacements are determined simultaneously and non-parametrically (directly) from the measured time series. Due to its simplicity and robustness, the approach adapted here for the OT calibration has been widely applied to time series analysis of various complex systems^8^.

## The power spectrum technique

We begin with the PS calibration method, which is basically a *linear* Langevin approach to the dynamics of the trapped bead. The idea of a Langevin model stems from the fact that the trapped particle dynamics is similar to a Brownian motion. Thus, the motion of the bead’s center of mass in a Hookean trapping potential is described by a linear Langevin equation^7^,

1 $$m\ddot{x}+\gamma \dot{x}-f(x)=\sqrt{2{k}_{B}T\gamma }{\rm{\Gamma }}(t)$$

where *f*(*x*) = −*kx* is the Hookean trapping force, and $-\,\gamma \dot{x}$ is the drag force exerted by the surrounding media with *γ* = 6*πηa*, *η*, and *a* being the drag coefficient, dynamic viscosity of the liquid, and the bead’s radius, respectively. Here, *x*(*t*) is the position of the particle at time *t*. *k*_*B*_, *T*, and the force Γ(*t*) are, respectively, the Boltzmann’s constant, temperature, and a random Gaussian white noise (Dirac delta correlated) that represents Brownian forces due to collisions with the surrounding molecules with the following mean and correlation function,

2 $$\langle {\rm{\Gamma }}(t)\rangle =\langle {\rm{\Gamma }}(t){\rm{\Gamma }}(t^{\prime} )\rangle =\delta (t-t^{\prime} \mathrm{).}$$

At low Reynolds numbers, one can ignore the inertial term in Eq. (1) to obtain

3 $$\gamma \dot{x}+kx=\sqrt{2{k}_{B}T\gamma }{\rm{\Gamma }}(t\mathrm{).}$$

It is interesting to note that *x*(*t*) in Eq. (3) represents a *Markov* process. This is due to the assumption that the random force Γ(*t*) is a memoryless process.

The Fourier transform of Eq. (3) results in a Lorentzian-type power spectrum,

4 $$p(f)=\frac{{k}_{B}T}{2{\pi }^{2}\gamma ({f}^{2}+{f}_{c}^{2})}=\frac{{D}_{SI}}{2{\pi }^{2}({f}^{2}+{f}_{c}^{2})},$$

where *p*(*f*) = 〈|*x*(*f*)|^2^〉/*L*, with *L* and *x*(*f*) being the size of the measured data, and the Fourier transform of the time series *x*(*t*), respectively. In addition, *f*_*c*_ = *k*/2*πγ* and *D*_*SI*_ are the corner frequency and diffusion coefficient in the SI unit system, respectively. There are several corrections to this simplified Lorentzian power spectrum, including among others those for handling finite sampling frequency, aliasing, including various hydrodynamic effects^7^.

It should be noted that in a typical experiment the time series *x*(*t*) are recorded in a scale other than meters - in our case volts - which should then be converted to the length units. Therefore, fitting the power spectrum of the measured time series *x*(*t*) would provide both *k* and *D* in a non-SI unit. Assuming that the measured voltage and the real displacement of the bead in the trap are linearly related, one determines the conversion factor using

5 $$\beta (m/Volt)=\sqrt{{D}_{SI}({m}^{2}/s)/{D}_{V}(Vol{t}^{2}/s)}$$

with *D*_*V*_ being the diffusion coefficient in the non-SI scale^7^. Our previous works^9^ have shown that, for the bead size used in this research, the linear behavior holds for the displacement range considerably larger than the Brownian amplitude of the bead in the trap.

**Figure 2:**
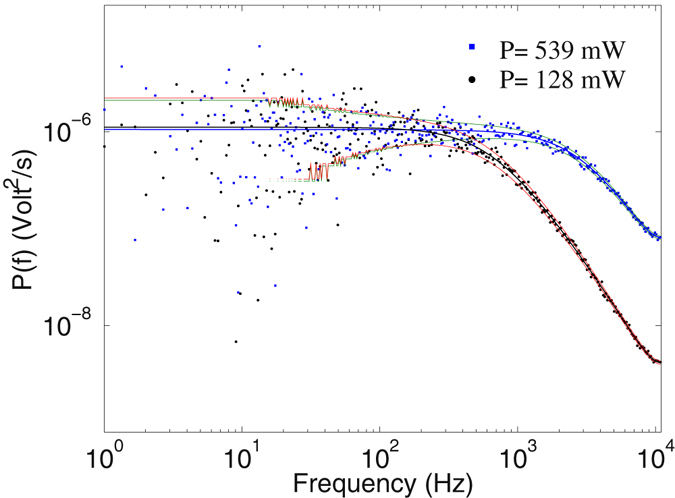
The power spectra of a trapped spherical polystyrene bead. Typical power spectra of a spherical polystyrene bead of diameter ~1 *μ*m, trapped by using laser powers of 128 mW and 539 mW. The solid lines represent fits to Eq. (4), with one *σ* confidence intervals using the calibration program of ref. 7. The time series *x*(*t*) for the spatial positions were recorded with sampling rate of 22 kHz.

Figure 2 shows typical power spectra of a polystyrene bead with a diameter of ~1 *μ*m, when trapped using laser powers of 128 mW and 539 mW, where we have included the hydrodynamic corrections using the calibration program provided by ref. 7. Within 1*σ* confidence interval there is no difference between the hydrodynamical corrected and uncorrected power spectra. The solid lines represent the fit to Eq. (4), which gives rise to corner frequencies (trap stiffnesses) of 558 ± 12.3 Hz (31.7 fN/nm) and 2216 ± 71 Hz (126 fN/nm), respectively. The non-SI diffusion coefficients in the aforementioned powers are read off, respectively, as *D*_*V*_ = 8.8 volt^2^/s and *D*_*V*_ = 129.9 volt^2^/s, implying conversion factors of *β* = 226 nm/volts and 59 nm/volts, respectively.

We note that it is assumed in the PS calibration method that, first, the restoring force is Hookean and, second, the diffusion coefficient of the bead is constant everywhere inside the optical trap. In what follows we present a nonlinear method, referred to as the Kramers-Moyal (KM) approach, for reconstructing the particle’s dynamical equation directly from its time series, which provides us with the spatial-dependence of the restoring force and the diffusion coefficient of a trapped particle.

## The Kramers-Moyal approach

For a stochastic system exhibiting Markov properties, the workflow of the KM approach involves the following steps^8^:

- Evaluate Markov properties and estimate the Markov-Einstein time scale, and write down the KM expansion for the dynamics of the probability distribution function.
- Verify the Pawula theorem that states that the KM expansion of the (marginal and conditional) probability distribution can be truncated after the second (diffusive) term, provided that the fourth-order KM coefficient *D* ^(4)^(*x*) vanishes.
- Compute the KM coefficients using the time series.
- Construct the Langevin equation with the computed drift function and diffusion coefficient.

### Markov-Einstein time scale

First, we check whether the recorded dynamics of the trapped bead follows a Markov process. In practice, a given dynamical process, such as movement of a bead in an optical trap, may have a finite Markov-Einstein (ME) time scale *τ*_*M*_ - the minimum time interval over which the time series *x*(*t*) can be considered a Markov process^8, 10–13^. Thus, let us estimate *τ*_*M*_ for a given time series.

The fundamental quantities related to the Markov processes are the conditional probability density functions (PDF). The PDF *p*(*x*_2_, *t*_2_|*x*_1_, *t*_1_) is defined by, $p({x}_{2},{t}_{2}|{x}_{1},{t}_{1})=\frac{p({x}_{2},{t}_{2};{x}_{1},{t}_{1})}{p({x}_{1},{t}_{1})}$, where *p*(*x*_2_, *t*_2_;*x*_1_, *t*_1_) is the joint PDF describing the probability of finding simultaneously *x*_1_ at time *t*_1_, and *x*_2_ at time *t*_2_. An important simplification made for a Markov process is that, the conditional multivariate joint PDF is written in terms of the products of simple two-parameter conditional PDFs^14^ via

6 $$p({x}_{N},{t}_{N};{x}_{N-1},{t}_{N-1};\cdots ;{x}_{2},{t}_{2}|{x}_{1},{t}_{1})=\prod _{i=2}^{N}p({x}_{i},{t}_{i}|{x}_{i-1},{t}_{i-1}).$$

This means that in the Markov process the ability to predict the value of *x*_*N*_ will not be enhanced by knowing its values in the steps prior to the most recent one. To investigate whether the underlying time series is a Markov process, one should test Eq. (6). But, doing so in practice for large values of *N* is well beyond the current computational capabilities. For *N* = 3 (three points or events), however, the condition will be

7 $$p({x}_{3},{t}_{3}|{x}_{2},{t}_{2};{x}_{1},{t}_{1})=p({x}_{3},{t}_{3}|{x}_{2},{t}_{2})$$

which should hold for any value of *t*_2_ in the interval *t*_1_ < *t*_2_ < *t*_3_. A process is then Markovian if Eq. (7) is satisfied for a *certain* time separation *t*_3_ − *t*_2_, in which case we define the ME time scale by *τ*_*M*_ = *t*_3_ − *t*_2_. For simplicity, we let Δ = *t*_2_ − *t*_1_ = *t*_3_ − *t*_2_. Thus, to compute *τ*_*M*_ we use a fundamental theorem of probability according to which we write any three-point PDF in terms of the conditional probability functions,

8 $$p({x}_{3},{t}_{3};{x}_{2},{t}_{2};{x}_{1},{t}_{1})=p({x}_{3},{t}_{3}|{x}_{2},{t}_{2};{x}_{1},{t}_{1})p({x}_{2},{t}_{2};{x}_{1},{t}_{1})$$

Using the properties of Markov processes to substitute Eq. (8), we obtain

9 $${p}_{{\rm{Mar}}}({x}_{3},{t}_{3};{x}_{2},{t}_{2};{x}_{1},{t}_{1})=p({x}_{3},{t}_{3}|{x}_{2},{t}_{2})p({x}_{2},{t}_{2};{x}_{1},{t}_{1})$$

Next, we introduce a *χ* ^2^-test to estimate the ME time scale by ref. 8

10 $${\chi }^{2}({\rm{\Delta }})=\int d{x}_{1}d{x}_{2}d{x}_{3}{[p({x}_{3},{t}_{3};{x}_{2},{t}_{2};{x}_{1},{t}_{1})-{p}_{{\rm{Mar}}}({x}_{3},{t}_{3};{x}_{2},{t}_{2};{x}_{1},{t}_{1})]}^{2}/[{\sigma }_{3-{\rm{joint}}}^{2}+{\sigma }_{{\rm{Mar}}}^{2}]$$

${\sigma }_{3-{\rm{joint}}}^{2}$ and ${\sigma }_{{\rm{Mar}}}^{2}$ are the errors of *p*(*x*_3_, *t*_3_; *x*_2_, *t*_2_; *x*_1_, *t*_1_) and *p*_M*ar*_(*x*_3_, *t*_3_; *x*_2_, *t*_2_; *x*_1_, *t*_1_), respectively. One then takes, *t*_1_ = 0 and *t*_2_ = *t*_3_/2 and plots the reduced *χ* ^2^, ${\chi }_{\nu }^{2}={\chi }^{2}/N$, where *N* is the number of degrees of freedom, as a function of the time scale Δ. The minimum value of ${\chi }_{\nu }^{2}$ corresponds to the best estimate of the ME time scale Δ = *τ*_*M*_. One can also use the Wilcoxon test to estimate the ME time scale for a given one-dimensional data set^8^.

### The Kramers-Moyal expansion

For a Markov process, knowledge of *P*(*x*, *t*|*x*_0_, *t*_0_) and *P*(*x*_0_, *t*_0_) suffices for generating the entire statistics of the data set, encoded in the *n*–point PDF, which satisfies a master Equation^14^, and is put in the form of a KM expansion:

11 $$\frac{\partial }{\partial t}P(x,t|{x}_{0},{t}_{0})=\sum {(-\frac{\partial }{\partial x})}^{k}[{D}^{(k)}(x,t)P(x,t|{x}_{0},{t}_{0})].$$

The KM coefficients *D* ^(*k*)^(*x*, *t*) are defined in terms of the conditional moments *M* ^(*k*)^(*x*, *t*):

12 $${D}^{(k)}(x,t)=\frac{1}{k!}\mathop{\mathrm{lim}}\limits_{{\rm{\Delta }}t\to 0}{M}^{(k)},$$

13 $${M}^{(k)}=\frac{1}{{\rm{\Delta }}t}\int dx^{\prime} {(x^{\prime} -x)}^{k}P(x^{\prime} ,t+{\rm{\Delta }}t|x,t\mathrm{).}$$

Though for a general stochastic process all the KM coefficients could be nonzero, according to Pawula’s theorem, the KM expansion could be truncated after the second term if the fourth-order coefficient *D* ^(4)^ vanishes or is very small^14^. In the present case, comparison of the KM coefficients for the normalized *x*(*t*) reveals that (${D}^{\mathrm{(4)}}/{D}^{\mathrm{(2)}})\simeq {10}^{-3}$, which justifies keeping only the first two KM coefficients. Therefore, the KM expansion reduces to a Fokker-Planck (FP) equation, i.e., Eq. 11 with *D* ^(*k*)^(*x*, *t*) = 0 for *k* ≥ 3. We note that non-vanishing higher order KM coefficients *D* ^(*k*)^(*x*, *t*) with *k* ≥ 3 have implication for the presence of jump events in the time series^15^.

### The Kramers-Moyal coefficients

If stationarity is given, then the KM coefficients *D* ^(*k*)^(*x*, *t*) are not explicitly time dependent. Therefore, the ensemble average in Eq. (13) can be estimated as conditional temporal average over the entire time series. To do so the state space of the process is discretized and the conditional average is calculated for every *x* (with some binning) separately. Therefore, one determines the *x*-dependent KM coefficients. In the case that the fourth-order coefficient *D* ^(4)^(*x*) vanishes, we need to estimate only *D* ^(1)^(*x*) and *D* ^(2)^(*x*), the drift function and diffusion coefficients.

### The Langevin dynamics

We note that the Fokker-Planck equation is in turn equivalent to the following Langevin equation (using Itô’s interpretation of stochastic integrals)^14^:

14 $$\frac{d}{dt}x(t)={D}^{(1)}(x)+\sqrt{2{D}^{(2)}(x)}\Gamma (t),$$

where Γ(*t*) is a random “force” with zero mean and Gaussian statistics, *δ*-correlated in time, i.e., 〈Γ(*t*)Γ(*t*′)〉 = *δ*(*t* − *t*′). Furthermore, Eq. (14) separates the deterministic - the first term, the drift - and the stochastic - the second term, diffusion - components of *x*(*t*) in terms of *D* ^(1)^ and *D* ^(2)^, respectively. Note that *D* ^(1)^ and *D* ^(2)^ are in general *position dependent*, but in the case of linear dependence of the drift term and a constant diffusion term (linear theory) Eq. (14) reduces to Eq. (3) with

15 $${D}^{(1)}(x)=-\frac{k}{\gamma }x\quad {D}^{(2)}(x)=\frac{{k}_{B}T}{\gamma }.$$

## Reconstruction of stochastic processes with given drift and diffusion coefficients

**Figure 3:**
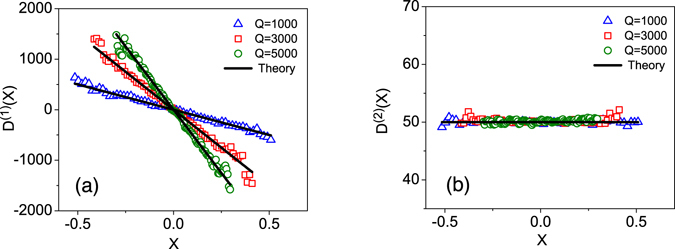
Reconstruction of a linear Langevin process. The KM-based estimated drift (**a**) and diffusion (**b**) terms for the synthetic time series with *Q* = 1000, 3000, and 5000, and *D*_0_ = 50. The solid lines in (**a**,**b**) show the considered values of *Q*s and *D*_0_. The analyzed time series consists of 10^7^ data points with a sampling interval of Δ*t* = 10^−6^. For *Q* = 5000 we averaged the results for the diffusion coefficients over 10 realisation of time series.

We consider a Langevin process with *D* ^(1)^(*x*) = −*Qx* and *D* ^(2)^(*x*) = *D*_0_ in Eq. (14), with *D*_0_ = 50 and the force constants, *Q* = 1000, 3000, and 5000. They are selected to be comparable with the experimental values. Synthetic time series were generated by numerical simulation of the corresponding dynamical equation using the Euler scheme^8^. Then, the KM coefficients *D* ^(1)^(*x*) and *D* ^(2)^(*x*) of the time series were estimated and plotted, respectively, in Fig. (3a,b) as functions of the position. The solid lines represent the values of *Q* and *D*_0_ as functions of *x*. Note that the estimated data points correctly follow the functions, hence confirming that our model can be safely used for a system with linear dynamics.

**Figure 4:**
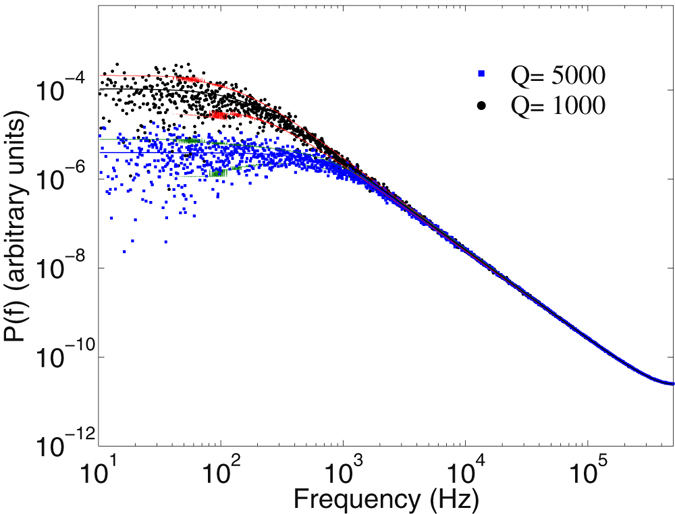
The power spectra of a linear Langevin process. The power spectra of the synthetic time series by the Langevin equation with *Q* = 1000 and 5000 and constant diffusion coefficient. The solid lines represent fits to Eq. (4). The computed values were *Q* = 1046 ± 36, 5089 ± 58, and *D*_0_ = 50.6 ± 0.1, 50.9 ± 0.5, respectively.

For comparison, the synthetic time series were also analyzed using the PS method, with the resulting power spectra of two of the cases depicted in Fig. (4). The results are, respectively, *Q* = 1046 ± 36, 2950 ± 43, and 5089 ± 58, and *D*_0_ = 50.6 ± 0.1, 50.4 ± 0.3, and 50.9 ± 0.5, which are also in very good agreement with the preset values, demonstrating that PS method can be correctly used for such linear systems.

**Figure 5:**
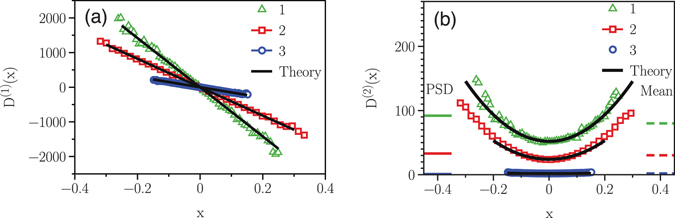
Reconstruction of a non-linear Langevin process. The KM-based estimated drift function (**a**) and diffusion coefficient (**b**) for the synthetic time series with *D* ^(1)^(*x*) = −*Qx* and position-dependent diffusion coefficient, *D* ^(2)^(*x*) = *D*_0_ + *bx* ^2^. Cases (1), (2) and (3) represent the parameter sets (*Q*, *D*_0_, *b*) ≡ (7100, 52, 1040), (4100, 24, 506) and (1500, 2, 28), respectively. The diffusion coefficient estimated by PS method and the KM average value are shown as small horizontal colored lines in panel (b).

Now, let us consider a Langevin process with a Hookean force, *D* ^(1)^(*x*) = −*Qx* and a variable diffusion coefficient, such as *D* ^(2)^(*x*) = *D*_0_ + *bx* ^2^. Three sets of the parameters (*Q*, *D*_0_, *b*) were chosen to be (7100, 52, 1040), (4100, 24, 506) and (1500, 2, 28), so selected to be comparable with the experimental values (see below). The resulting KM coefficients *D* ^(1)^(*x*), and *D* ^(2)^(*x*) are shown in Fig. (5a,b), respectively. The solid lines show the set functionalities. It is clear that the estimated values follow accurately the expected functions, confirming that the proposed method is able to capture important features of nonlinear systems.

**Figure 6:**
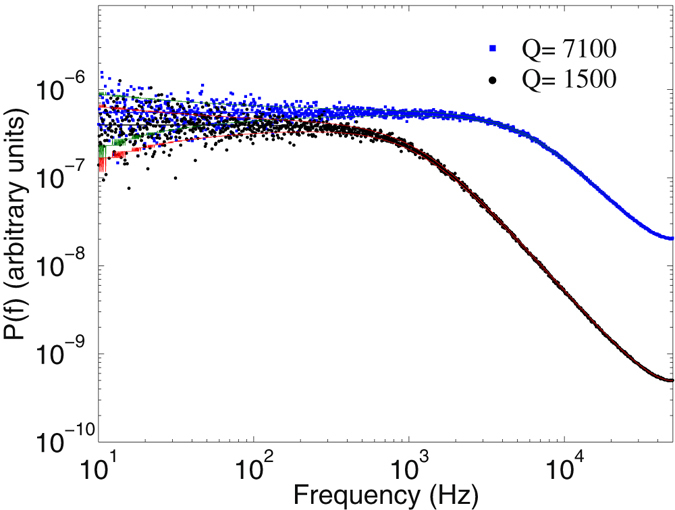
The power spectra of a non-linear Langevin process. The power spectra of the synthetic time series by the Langevin equation with *D* ^(1)^(*x*) = −*Qx* and position-dependent diffusion coefficient, *D* ^(2)^(*x*) = *D*_0_ + *bx* ^2^, with the parameter sets of (*Q*, *D*_0_, *b*) ≡ (1500, 2, 28) and (7100, 52, 1040), respectively. Solid lines represent the fit to Eq. (4), which yields (1564 ± 6, 2.2 ± 0.1, NA) and (8629 ± 13, 92.0 ± 1.9, NA), respectively.

Similar to the previous case, for comparison with our theory the synthetic time series were also analyzed using the PS method, Fig. (6), which yielded (8629 ± 13, 92.0 ± 1.9, NA), (4561 ± 9, 34.0 ± 1.4, NA) and (1564 ± 6, 2.2 ± 0.1, NA), respectively.

Note that the PS method cannot provide an estimate of the coefficient *b*, as it assumes that the diffusion coefficient is constant. In fact, the PS method estimates the diffusion coefficient to be very close to its average value. For comparison, both values are shown as small colored lines in Fig. (5b).

## Results

### Results of the Kramers-Moyal approach

Next, we present the results of KM method for the measured data at nine laser powers, from 73 mW to 539 mW. Once a bead is trapped, 10 × 3 seconds positional time series *x*(*t*) were recorded with sampling rate of 22 kHz (see section Methods). Two typical power spectra of the recorded data are shown in Fig. (2).

**Figure 7:**
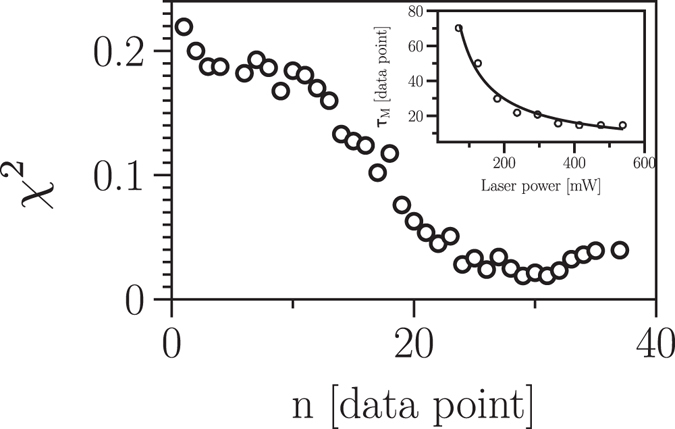
The Markov-Einstein time scale. Estimation of the Markov-Einstein time scale using a *χ* ^2^ test, Eq.(10). The laser power dependence of the time scale is plotted in the inset that scales with the laser intensity *I* as ~*I* ^−0.9^.

Let us first check whether the recorded time series represents a Markov process and, if so, determine their Markov-Einstein time scale *τ*_*M*_. Figure 7 shows the *n* = Δ/*dt* (*dt* = 1/22000 s)-dependence of the ${\chi }_{\nu }^{2}$ for a typical time series of a bead trapped at the laser power of 128 mW. The ME time scale *τ*_*M*_ for all the recorded time series was estimated to be about 5 to 70 data points (227–3181 *μ*s). We found that the dynamics of the trapped particle at larger laser powers has a smaller ME time scale. The laser power dependence of ME time scale is plotted in the inset of Fig. 7 and *τ*_*M*_ scales with laser intensity *I* as ~*I* ^−0.9^.

**Figure 8:**
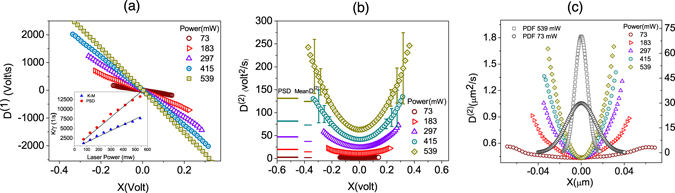
Disentangling stochastic characteristics of particles trapped in optical tweezers. Experimental results: The drift (**a**) and diffusion coefficients (**b**) for 5 laser powers. The drifts estimated by the PS method are embedded in the inset of (**a**). The small colored lines in (**b**) show the constant diffusion coefficients estimated by the PS method, as well as the average diffusion coefficients. (**c**) The graphs in (**b**) re-plotted after converting the position to the SI unite using Eq. (5) with *D*_*V*_ being the intercept of the relevant graph. The gray and black graphs show the probability distribution in the presence of the bead as a function of the position for the lowest and highest laser powers.

The drift function *D* ^(1)^(*x*) and the diffusion coefficients *D* ^(2)^(*x*) of the recorded time series were calculated using Eq.(12). The results are summarized in Fig. 8. For each laser power, the drift and diffusion functions were averaged over 10 ensembles. The error is large for larger *x*, as shown in, for instance, Fig. 8b.

As shown in Fig. (8a), the drift coefficients are linear functions of *x*, confirming the Hookean nature of the trapping force. The slope of the graphs are plotted against the laser power in the inset of the figure, which also indicates an almost linear dependence, a hallmark of an OT. All the time series were also analyzed using the PS method; the results are embedded in the inset of Fig. (8a). One can easily see that the stiffness estimated by the PS method deviates (overestimates) from that provided by the KM method, which is more pronounced for larger laser powers.

**Figure 9:**
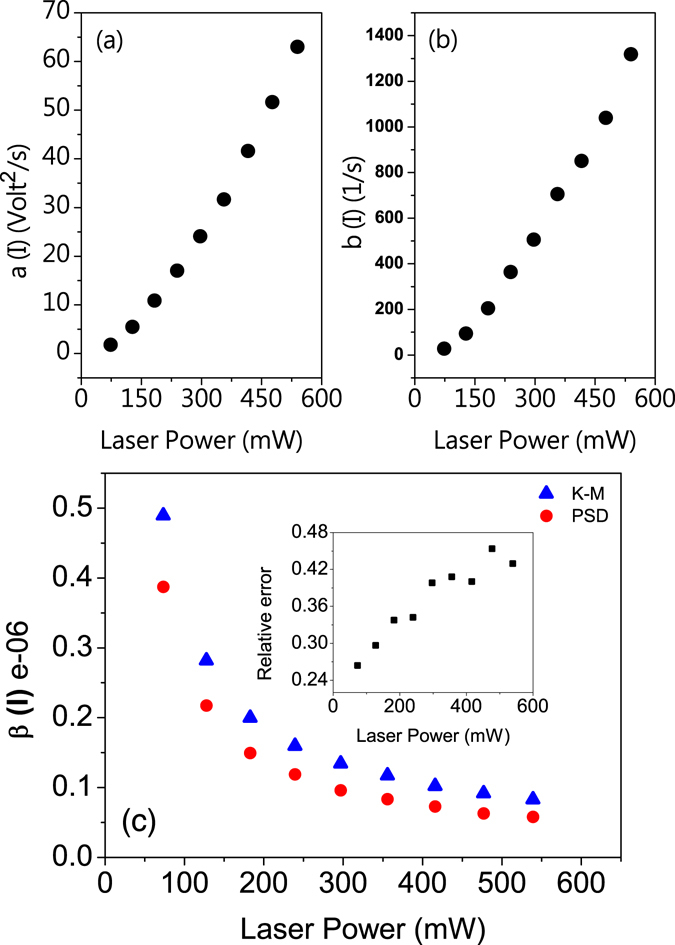
Intensity-dependence of the Diffusion coefficient. Intensity-dependence of the coefficients (**a**) *a*(*I*), (**b**) *b*(*I*), and (**c**) the conversion factor *β*, resulted from the KM and PS methods. The inset shows the relative error for the PS method as a function of laser power. The coefficient *b*(*I*) scales with intensity as *I* ^1.5^ for large intensities.

Figure (8b) presents the resulting diffusion coefficient *D* ^(2)^(*x*) as a function of the position for various laser powers, indicating a nonlinear (fairly quadratic) behavior. For each case, the value of the diffusion coefficient estimated by the PS method, as well as the KM average values are shown by colored line next to the graphs. It can be seen that the estimates by the PS method are very close to those of the average values. Figure (8c) shows the same graphs presented in Fig. (8b) after converting the horizontal axis to the SI unit using Eq. (5) with *D*_*V*_ being the intercept of the relevant graph (see below). Note that using *D*_*V*_ provided by the PS method would introduce considerable errors in *β*, and underestimate the real displacement. This artifact could affect any OT-based experiments that demands accurate displacement measurement. To ensure that the bead was likely to be in the nonlinear diffusion regime, we also calculated the PDF of the position, two extreme cases of which are included in Fig. (8c) as the black and grey graphs. They confirm that the bead has spent reasonable amounts of time at positions with larger diffusion coefficients. Furthermore, the graphs in Fig. (8b) were fitted to the quadratic function, *D* ^(2)^(*x*, *I*) = *a*(*I*) + *b*(*I*)*x* ^2^, with *a* and *b* as the free parameters (*x* has the unit of volt). The resulting values of *a* and *b* as functions of the laser power are summarized in Fig. (9a,b), respectively. It is evident that only at very low laser powers the parameter *b* would be negligible, in which case the parameters provided by the PS method would be valid. The conversion factors provided by the KM and PS methods as a function of laser power are presented in Fig. (9c). Once again, it is clear that there is discernible differences between the KM and PS results. The inset of the figure shows the relative error generated by the PS estimation, which always underestimates the conversion factor.

Table 1 summarizes all the results obtained by the KM and PS analyses. As the table indicates, the PS method always overestimates the stiffness and underestimates the conversion factor. It is worth mentioning that the estimated force based on the PS calibration could, however, have smaller error compared to the measured stiffness or displacement, as the two errors can partially cancel out.

**Table 1:** Comparison of results from PS and KM methods.

Laser power (mW)	*D*_*v*_ (PS & KM) *Volt* ^2^/*s*	k (PS & KM) *fN*/*nm*	*β* × 10^−8^ (PS & KM)	RelDif of *β*%	RelDif of *K*%	RelDif of *D*_*v*_%
73	2.9 ± 0.1 & 1.8 ± 0.1	17.7 ± 0.5 & 11.3 ± 0.3	39.8 & 49.0	23	36	38
128	8.8 ± 0.2 & 5.5 ± 0.1	31.7 ± 0.7 & 19.4 ± 0.4	22.6 & 28.1	24	39	38
183	18.9 ± 0.2 & 10.9 ± 0.2	47.3 ± 1.2 & 28.9 ± 0.6	15.9 & 20.0	26	39	42
239	30.7 ± 0.3 & 17.0 ± 0.2	61.9 ± 2.0 & 37.4 ± 0.7	12.1 & 16.0	32	40	45
297	45.1 ± 0.5 & 24.1 ± 0.5	74.4 ± 1.2 & 45.0 ± 0.9	10.0 & 13.4	34	40	47
356	61.9 ± 1.2 & 31.7 ± 0.6	87.7 ± 2.2 & 52.0 ± 1.0	8.6 & 11.7	36	41	49
415	81.4 ± 1.3 & 41.7 ± 0.8	98.2 ± 2.5 & 58.2 ± 1.2	7.5 & 10.2	36	41	49
477	104.0 ± 1.3 & 51.7 ± 1.0	110.3 ± 1.7 & 64.1 ± 1.3	6.6 & 9.2	40	42	50
539	129.9 ± 4.1 & 63.0 ± 1.3	125.9 ± 4.2 & 72.1 ± 1.4	5.9 & 8.3	41	43	51

**Figure 10:**
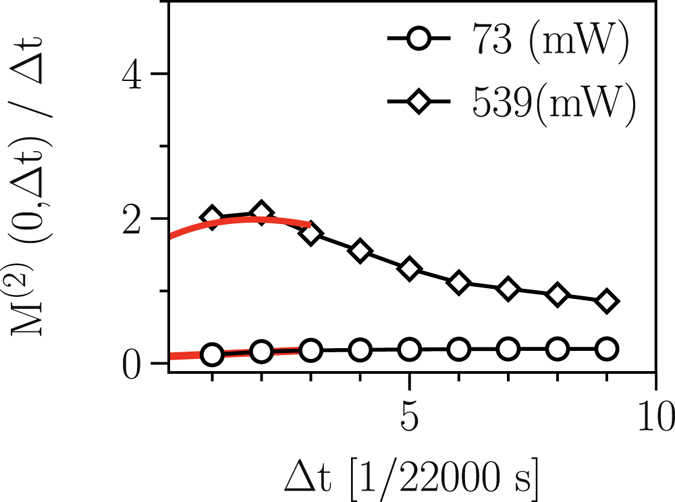
Non-diverging behavior of diffusion coefficient. The limit Δ*t* → 0 of *M* ^(2)^(*x* = 0, Δ*t*)/Δ*t* (the diffusion coefficients) for two laser powers 73 mW and 539 mW. This indicates finite values of the diffusion coefficients in that limit (red curves).

One might argue that the quadratic position-dependence of the diffusion coefficient *D* ^(2)^(*x*) may arise from the finite sampling rates. In order to check that possibility, we note that considering the definition of Eq. (13) and holding terms up to the order of Δ*t* ^2^, the first and second conditional moments would have the following expression for *M* ^(1)^(*x*, Δ*t*) and *M* ^(2)^(*x*, Δ*t*)^8, 16^,

16 $$\begin{array}{rcl}{M}^{\mathrm{(1)}}(x,{\rm{\Delta }}t) & \approx  & {\rm{\Delta }}t{D}^{\mathrm{(1)}}(x)+\frac{{\rm{\Delta }}{t}^{2}}{2}[{D}^{\mathrm{(1)}}({D}^{\mathrm{(1)}})^{\prime} +{D}^{\mathrm{(2)}}({D}^{\mathrm{(1)}})^{\prime\prime} ]+O({\rm{\Delta }}{t}^{3})\\ {M}^{\mathrm{(2)}}(x,{\rm{\Delta }}t) & \approx  & 2{\rm{\Delta }}t{D}^{\mathrm{(2)}}(x)+{\rm{\Delta }}{t}^{2}[({D}^{\mathrm{(1)}}{)}^{2}+2{D}^{\mathrm{(2)}}({D}^{\mathrm{(1)}})^{\prime} \\  &  & +{D}^{\mathrm{(1)}}({D}^{\mathrm{(2)}})^{\prime} +{D}^{\mathrm{(2)}}({D}^{\mathrm{(2)}})^{\prime\prime} ]+O({\rm{\Delta }}{t}^{3}\mathrm{).}\end{array}$$

where the first terms yields the drift and diffusion coefficients in the limit Δ*t* → 0. For finite Δ*t*, however, the KM coefficients will be subject to some corrections, and the expansion will be meaningful if the term of the order of Δ*t* is enough small. One possible way of avoiding the artifact of sampling interval in the estimation of the drift and diffusion coefficients is plotting *M* ^(1,2)^(*x*, Δ*t*)/Δ*t* for fixed *x* versus Δ*t*, and taking the limit Δ*t* → 0^8^. In Fig. (10), we plot *M* ^(2)^(*x* = 0, Δ*t*)/Δ*t* in the limit of Δ*t* → 0 for two laser powers, namely, 73 mW and 539 mW, which indicates finite values of the diffusion coefficients in that limit.

The physical picture behind the spatial dependence of the diffusion coefficient can be very complex. In general, one may argue that the spatial dependence of the diffusion coefficient could be due to the changes in the quantities that parameterize the interaction of the bead with the surrounding medium and the electric field of the laser beam. One possible reason could be as follows. It is known that when a polystyrene sulphate sphere is dispersed in water, the ionic groups bonded to their surface dissociate, giving rise to a screened electrostatic interaction. In this case, the bead will possess effective negative charge *Z* ^*^, screened by the same amount of positive charges over the Debye-Huckel screening length^17^. In the static limit, the negatively-charged bead with its positive shell will have zero electric dipole. In the presence of temperature, however, a random force (which can be considered as a white noise in ME time scale) due to the environment (which is responsible for Brownian motion of the bead) will act on the bead and its positive shell. The random collisions play two roles: first, they exert a random “push” on the bead and, second, they disturb the symmetry of the charge distribution around the bead, which induces a time-dependent contribution to the electric dipole moment of the bead with a random orientation. In our case (for the bead with radius of $R\simeq 0.5\,\mu {\rm{m}}$) *Z* ^*^ is about 5 × 10^3^ *e* C and $|{\bf{P}}|\simeq 7.5\times {10}^{-26}$ C m (by considering that the charge separation is of order of 1 *Å*)^17^.

The radial force acting on the electric dipole in the presence of the laser electric field (${\bf{E}} \sim {E}_{x}{\bf{x}}$) is given by, ${F}_{x}=\frac{\partial ({P}_{x}{E}_{x})}{\partial x}$, which has a zero mean due to the rapid fluctuations of the electric field and random orientations of the total dipole moment *P*_*x*_. The random collisions due to the thermal fluctuations disturb the symmetry of the charge distribution around the bead, and induce a time-dependent electric dipole moment with a random orientation. The presence of the electric field of the laser can further alter the produced dipole. In linear optics, the polarisation of the charge distribution depends on the strength of the applied electric field and can be described by, *P*_*x*_ = *P*_0*x*_ + *χE*_*x*_, where $\chi =\frac{\partial {P}_{x}}{\partial {E}_{x}}$ is known as the linear optical susceptibility. For a Gaussian laser beam with beam waist of *w*_0_, the force will have a variance given by, $\langle {|{F}_{x}|}^{2}\rangle =(\frac{{x}^{2}}{2{w}_{0}^{4}}){E}_{0}^{2}{({P}_{0x}+2\chi {E}_{0})}^{2}$. In order to account for this force one must add a force such as $\sqrt{\langle {|{F}_{x}|}^{2}\rangle }\sqrt{{\tau }_{M}}/\gamma \,f(t)$ to the linear Langevin Equation, Eq. (14). This force can be accounted for by an extra diffusion coefficient, ~*bx* ^2^ with $b=1/2{({P}_{0x}+2\chi \sqrt{I})}^{2}{I}^{0.1}/(2{w}_{0}^{4}{\gamma }^{2})$. We note that the polarisation *P*, induced by the charge separation and ME scale *τ*_*M*_ (as shown in Fig. 7) are intensity-dependent described by ~*I* and ${\tau }_{M} \sim {I}^{-0.9}$, respectively. One then finds that *x*-dependent part of *D* ^(2)^ scales as ${I}^{2}\times {I}^{-0.9} \sim {I}^{1.1}$, which has super-linear scaling. The observed value for the exponent is $\simeq 1.5$ for large intensities (Fig. 9b).

One might also argue that the rapid fluctuations in the intensity of the trapping laser could contribute to the measured diffusion coefficients in two ways: first, by inducing fluctuation in the measured trap stiffness and, second, by contributing to the measured diffusion coefficients with a second-order correction in the position relative to the origin. In order to make sure that what we see is not an artifact induced by the fluctuation in the intensity of the laser power, the same procedure was applied to the normalised positional data (positional series divided by the total intensity), and the results indicate again similar position-dependent diffusion coefficients. Further, we measured the total intensity of the trapping laser, both directly at the exit of the laser source and at the QPD position when the trap is empty (Z-signal of the QPD). The histogram of the resulting data was perfectly Gaussian (with standard deviation of 1%) at the tuned power (data are not shown).

## Summary

Linear techniques, such as the spectral and correlation analysis, can uncover only the linear structures of time series. We showed that the time series recorded from the movements of a bead in an optical trap contain nonlinear components and, therefore, one needs a nonlinear approach for their analysis. We used a nonlinear approach to stochastic analysis that enables us to determine the stochastic dynamic equation of a trapped particle. The approach is based on the estimations of the force constant and the diffusion coefficient based on the Kramers-Moyal conditional moments, providing us with position-dependence physical parameters. We discovered that the diffusion coefficient depends almost quadratically on the position, in contrast with the assumption of the power spectrum method. Our detailed analysis of the measured time series reveals that the power spectrum analysis overestimates considerably the force constant and underestimates the conversion factor.

We believe that the present study has important implications for the OT-based applications, and in particular for single-molecule studies. For instance, by accurate estimation of the force constant, our proposed approach allows for reliable estimation of the forces and mechanical work associated with unfolding and refolding of biomolecules^18–20^.

## Methods

### Experimental setup

Our OT setup consists of a Nd:YAG laser (Coherent, *λ* = 1064 nm), focused using a water immersion objective (Olympus) in an optimal condition^21, 22^. A quadrant photodiode (Hamamatsu, S5980) is positioned at the Back-Focal-Plane (BFP) of the condenser, which allows for very accurate detection of the displacements of a trapped bead through the BFP detection scheme^23^. The voltages of the QPD were first amplified and then digitized using an A/D card (National Instruments). The polystyrene beads were purchased from Bangs Lab. with a mean diameter of ~1 *μ*m. Once a bead is trapped, 10 × 3 seconds positional time series *x*(*t*) were recorded with sampling rate of 22 kHz. All the trapping experiments were conducted under the optimal condition with almost zero aberrations at a depth of ~10 *μ*m, in order to be able to neglect the hydrodynamic effect of the chamber walls. The measurements were repeated at nine laser powers from 73 mW to 539 mW. Two typical power spectra of the recorded data are shown in Fig. (2).
